# Supplementary material for: Efficacy and safety of diazoxide for treating hyperinsulinemic hypoglycemia: A systematic review and meta-analysis
Source: PLoS One. 2021 Feb 11;16(2):e0246463. doi: 10.1371/journal.pone.0246463 (PMC7877589; doi:10.1371/journal.pone.0246463)
Supplement: S1 Table — (DOC) [file pone.0246463.s002.doc]

**Table S1 Search strategies for Embase.**

| **Period of Search** | **Search strategies** |
| --- | --- |
| June 2019 | ('hyperinsulinemic hypoglycemia':ab,ti OR 'congenital hyperinsulinemia':ab,ti) AND (children:ab,ti OR 'neonates':ab,ti OR 'infants':ab,ti) AND diazoxide:ab,ti |
