# Supplementary material for: Efficacy and safety of diazoxide for treating hyperinsulinemic hypoglycemia: A systematic review and meta-analysis
Source: PLoS One. 2021 Feb 11;16(2):e0246463. doi: 10.1371/journal.pone.0246463 (PMC7877589; doi:10.1371/journal.pone.0246463)
Supplement: Attachment — Submitted filename: Response Letter.doc [file pone.0246463.s004.doc]

Dear editor,

On behalf of all co-authors, we would like to express our great appreciation to you and the reviewers for your positive, constructive and insightful comments on our manuscript entitled “Efficacy and safety of diazoxide for treating hyperinsulinemic hypoglycemia: a systematic review and meta-analysis”. These comments and suggestions are really helpful in improving the quality of our manuscript. Accordingly, we have revised the manuscript. In addition, point-by-point responses to the editor’s and reviewers’ comments are listed below.

We, again, thank you and the reviewers for your constructive comments and hope that the revision is acceptable for publication in your journal.

We are looking forward to hearing from you soon.

Sincerely,

**Point-by-point responses to the editor’s comments:**

**Important Comments:**

**Comment 1:** Introduction The authors need to state the objective in the last paragraph, although it has been described in the Abstract.

**Response:** We thank you for this kind reminding. Accordingly, we have added it to Introduction in the newly revised version.

**Comment 2:** Study selection It will be informative to provide more details of search strategy used for Embase in appendix.

**Response:** As suggested, we have added it in the newly revised version (Table S1).

**Comment 3:** The authors have indicated clinical studies as an inclusion criteria. However, it has indicated in the abstract that cohort studies are included. The quality assessment use was the NOS checklist. Hence, the included studies were observational type, rather than clinical studies. Please, provide what type of observational studies....... Only cohort or/ and case-control designs. In Table 1, please add 1 more column for study design.

**Response:** Thanks for this kind suggestion, we have added it to Table 1 in the newly revised version.

**Comment 4:** Data extraction Please, indicate the initials of two authors who had done study selection/data extraction.

**Response:** Thanks for this kind suggestion, we have added them in the newly revised version.

**Comment 5:** Statistical analysis It is essential to describe the outcome measurement ( e.g OR or RR and its 95%CI). To be more informative, please define the response rate (e.g what is numerator/ denominator, etc).

**Response:** Thanks for this kind suggestion, we have added them to Statistical analysis in the newly revised version.

**Comment 6:** Reference. In addition to #21 (Lu et al).......... It will be better to refer Higgins et al 2019 (available from the Cochrane Library website).

**Response:** Thanks for this kind suggestion, we have added it to Reference in the newly revised version.

**Point-by-point responses to the reviewer’s comments:**

**Reviewer #1:**

**Important Comments:**

**Comment 1:** Overall, the manuscript was well written, and presented adequate study methodology, results and discussion. Some minor clarifications are required on the following.

**Response:** It is pleasing to have acknowledged our diligence in completed this manuscript. We appreciate the reviewer’s kindly comments.

**Comment 2:** Discussion Para 2, Line 2: “Its use has increased… having more babies than ever before.” – This statement is unclear.

**Response:** Thanks for this kind suggestion, we have rewritten them in the newly revised version.

**Comment 3:** Para 2, Line 9: “However, the exception is that children with ABCC8 mutation respond to diazoxide, indicating cellular adaptation and red blood cells”- The statement regarding red blood is unclear

**Response:** Thanks for this kind suggestion, we have rewritten them in the newly revised version.
