# Supplementary material for: Efficacy and safety of diazoxide for treating hyperinsulinemic hypoglycemia: A systematic review and meta-analysis
Source: PLoS One. 2021 Feb 11;16(2):e0246463. doi: 10.1371/journal.pone.0246463 (PMC7877589; doi:10.1371/journal.pone.0246463)
Supplement: Attachment — Submitted filename: Response to Reviewers.doc [file pone.0246463.s005.doc]

Dear editor,

On behalf of all co-authors, we would like to express our great appreciation to you for your positive and constructive comments on our manuscript entitled “Efficacy and safety of diazoxide for treating hyperinsulinemic hypoglycemia: a systematic review and meta-analysis”. These comments and suggestions are really helpful in improving the quality of our manuscript. Accordingly, we have revised the manuscript. In addition, point-by-point responses to the editor’s comments are listed below.

We hope that the revision is acceptable for publication in your journal.

We are looking forward to hearing from you soon.

Sincerely,

**Point-by-point responses to the editor’s comments:**

**Important Comments:**

**Comment 1:** The current analysis was based on six cohort studies. In the Background, six clinical studies. Please make a correction.

**Response:** Accordingly, we have corrected it in the newly revised version.

**Comment 2:** The inclusion criteria were as follows: (1) clinical studies focusing on ..................... This was incorrect. The authors have selected only cohort studies. No clinical studies. Plese update this. selection criteria. I have already highlighted this point in initial comments.

**Response:** Accordingly, we have corrected it in the newly revised version.

**Comment 3:** The authors still need substantial improvements in grammar/language throughout the text.

Examples,

In abstract

The meta-analysis of the efficacy and safety of diazoxidein treating HH was performed by searching the PubMed, Embase, ..............

Two missing words "relevant studies" between searching the and PubMed,

A total of 6studies, ................... It should be presented 'a total of six cohort studies'.,

Among the cohort studies, the pooled estimate of patients who were diazoxide responsive was 71% (95% CI 50%–93%.................. Please rephrase this sentence by adding pool estimates of what outcome.

In Text

A meta-analysis of cohorts based on the random-effects............................ It should describe 'cohort studies, instead of cohorts. .

**Response:** Accordingly, we have carefully proofread the manuscript and corrected the spelling and grammatical errors in the revised manuscript.
