# Supplementary material for: Efficacy and safety of diazoxide for treating hyperinsulinemic hypoglycemia: A systematic review and meta-analysis
Source: PLoS One. 2021 Feb 11;16(2):e0246463. doi: 10.1371/journal.pone.0246463 (PMC7877589; doi:10.1371/journal.pone.0246463)
Supplement: Attachment — Submitted filename: Response to Reviewers.doc [file pone.0246463.s006.doc]

Dear editor,

On behalf of all co-authors, we would like to express our great appreciation to you for your positive and constructive comments on our manuscript titled “Efficacy and safety of diazoxide for treating hyperinsulinemic hypoglycemia: a systematic review and meta-analysis.” These comments and suggestions are really helpful in improving the quality of our manuscript. Accordingly, we have revised the manuscript. In addition, point-by-point responses to the editor’s comments are listed below.

We hope that the revision is acceptable for publication in your journal.

We look forward to hearing from you soon.

Sincerely,

**Point-by-point responses to the editor’s comments:**

**Important Comments:**

**Comment 1:** The current analysis was based on six cohort studies. In the Background, six clinical studies. Please make a correction.

**Response:** Thank you for the comments. We have corrected it in the revised version.

**Revised text: “**This meta-analysis based on six cohort studies was conducted and aimed to investigate the efficacy and safety of diazoxide for treating CHI.”

**Comment 2:** The inclusion criteria were as follows: (1) clinical studies focusing on ..................... This was incorrect. The authors have selected only cohort studies. No clinical studies. Please update this. selection criteria. I have already highlighted this point in initial comments.

**Response:** Thank you for the comments. We have corrected it in the revised version.

**Revised text:** “Cohort studies focusing on the efficacy and safety of diazoxide in treating HH.”

**Comment 3:** The authors still need substantial improvements in grammar/language throughout the text.

Examples,

In abstract

The meta-analysis of the efficacy and safety of diazoxidein treating HH was performed by searching the PubMed, Embase, ..............

Two missing words "relevant studies" between searching the and PubMed,

A total of 6studies, ................... It should be presented 'a total of six cohort studies'.,

Among the cohort studies, the pooled estimate of patients who were diazoxide responsive was 71% (95% CI 50%–93%.................. Please rephrase this sentence by adding pool estimates of what outcome.

In Text

A meta-analysis of cohorts based on the random-effects............................ It should describe 'cohort studies, instead of cohorts. .

**Response:** Thank you for the suggestion. We have carefully proofread the manuscript and corrected the spelling and grammatical errors in the revised manuscript.

**Revised text:** “The meta-analysis of the efficacy and safety of diazoxide in treating HH was performed by searching relevant studies in the PubMed, Embase, and Cochrane databases.”

“A total of 6 cohort studies, involving 1142 participants, met the inclusion criteria.”

“Among the cohort studies, the pooled estimate of the response rate of diazoxide therapy was 71%.”

“A meta-analysis of cohort studies based on the random-effects model was conducted to evaluate the clinical response rate.”

**Comment 4**: The authors have to update literature search whether there may be additional published studies after your initial search in 2019. This is crucially important in this field as the evidence must be based on comprehensive review of all eligible studies.

**Response:** Thank you for the suggestion. The literature search has been updated up to January 2021.

**Revised text:** “The PubMed, Embase, and Cochrane databases were searched for studies published up to January 2021.”

“The electronic search identified 348 studies. Three additional studies were found by hand searching from the reference lists of other review studies. According to the inclusion criteria, 227 studies remained after removing the duplicates. Subsequently, 193 irrelevant studies were excluded. Of the remaining 34 studies, 15 were letters, reviews, and meta-analyses, and hence excluded. The remaining studies were systematically reviewed, and 19 were qualified for full-text reading. After full-text reading, five studies not focusing on children, three lacking usable data, and five case reports were excluded.” (Figure 1)
